# Pathogen-Specific Impacts of the 2011–2012 La Niña-Associated Floods on Enteric Infections in the MAL-ED Peru Cohort: A Comparative Interrupted Time Series Analysis

**DOI:** 10.3390/ijerph17020487

**Published:** 2020-01-12

**Authors:** Josh Colston, Maribel Paredes Olortegui, Benjamin Zaitchik, Pablo Peñataro Yori, Gagandeep Kang, Tahmeed Ahmed, Pascal Bessong, Esto Mduma, Zulfiqar Bhutta, Prakash Sunder Shrestha, Aldo Lima, Margaret Kosek

**Affiliations:** 1 Division of Infectious Diseases and International Health, University of Virginia School of Medicine, Charlottesville, VA 22903, USA; josh.colston@virginia.edu; 2 Asociación Benéfica Prisma, Iquitos 16006, Peru; mparedeso@prisma.org.pe; 3 Department of Earth and Planetary Sciences, Johns Hopkins Krieger School of Arts and Sciences, Baltimore, MD 21218, USA; zaitchik@jhu.edu; 4 Division of Infectious Diseases and International Health, University of Virginia, Charlottesville, VA 22903, USA; pyori@virginia.edu; 5 Christian Medical College, Vellore 632004, India; gkang@cmcvellore.ac.in; 6 Nutrition & Clinical Services Division, International Centre for Diarrhoeal Disease Research, Bangladesh (icddr,b), Dhaka 1213, Bangladesh; tahmeed@icddrb.org; 7 University of Venda, Thohoyandou 0950, South Africa; Pascal.Bessong@univen.ac.za; 8 Haydom Global Health Institute, Haydom P.O. Box 9000, Tanzania; estomduma@gmail.com; 9 Department of Pediatrics and Child Health, Aga Khan University, Karachi 74800, Pakistan; zulfiqar.bhutta@aku.edu; 10 Department of Child Health, Institute of Medicine of Tribhuvan University, Kirtipur 44618, Nepal; prakashsunder@hotmail.com; 11 Federal University of Ceará, Fortaleza 60020-181, Brazil; alima@ufc.br

**Keywords:** climate change, diarrheal disease, infectious disease, ENSO, La Niña, flooding, natural disasters, enteric viruses, enteric bacteria, rotavirus

## Abstract

Extreme floods pose multiple direct and indirect health risks. These risks include contamination of water, food, and the environment, often causing outbreaks of diarrheal disease. Evidence regarding the effects of flooding on individual diarrhea-causing pathogens is limited, but is urgently needed in order to plan and implement interventions and prioritize resources before climate-related disasters strike. This study applied a causal inference approach to data from a multisite study that deployed broadly inclusive diagnostics for numerous high-burden common enteropathogens. Relative risks (RRs) of infection with each pathogen during a flooding disaster that occurred at one of the sites—Loreto, Peru—were calculated from generalized linear models using a comparative interrupted time series framework with the other sites as a comparison group and adjusting for background seasonality. During the early period of the flood, increased risk of heat-stable enterotoxigenic *E. coli* (ST-ETEC) was identified (RR = 1.73 [1.10, 2.71]) along with a decreased risk of enteric adenovirus (RR = 0.36 [0.23, 0.58]). During the later period of the flood, sharp increases in the risk of rotavirus (RR = 5.30 [2.70, 10.40]) and sapovirus (RR = 2.47 [1.79, 3.41]) were observed, in addition to increases in transmission of *Shigella* spp. (RR = 2.86 [1.81, 4.52]) and *Campylobacter* spp. (RR = 1.41 (1.01, 1.07). Genotype-specific exploratory analysis reveals that the rise in rotavirus transmission during the flood was likely due to the introduction of a locally atypical, non-vaccine (G2P[4]) strain of the virus. Policy-makers should target interventions towards these pathogens—including vaccines as they become available—in settings where vulnerability to flooding is high as part of disaster preparedness strategies, while investments in radical, transformative, community-wide, and locally-tailored water and sanitation interventions are also needed.

## 1. Introduction

Climate change is increasingly understood to represent an impending global public health threat, since numerous health outcomes are sensitive to meteorological patterns [1,2]. In addition to rising surface temperatures, variability in precipitation and evapotranspiration is set to increase in the coming decades, exaggerating the pattern of high rainfall at the equator and polar fronts and low rainfall across the subtropical heights [3]. The impacts of changing rainfall patterns on health will likely not be linear but felt most pronouncedly at the extremes, with both heavy precipitation events and more frequent droughts making water sources and food systems more precarious and disease and injury more likely [4,5,6]. Floods, already the most common type of national disaster, are likely to increase in frequency and severity in many regions as climate change brings about sea level rises and shifts in rainfall patterns [7,8,9]. There is particular reason for concern in the Amazon, where La Niña conditions are associated with major floods, including the record floods of 2012 [10,11], and where recent intensification of flood events may be the result of a climate change induced trend towards La Niña, such as sea surface temperature patterns [12].

The health impacts of floods are manifold and include injury and drowning, toxic exposure, and increased transmission of vector- and waterborne diseases [9], but enteric disease outbreaks are one of the most widely recognized [6]. Following heavy precipitation events, floodwater and surface runoff may overwhelm drainage and wastewater systems, causing the dispersal of enteric viruses, bacteria and protozoa through the environment and the contamination of surface and groundwater supplies as well as crops [6,7,13,14,15]. Those most vulnerable to these hazards will be populations in low-resource countries, as rapid, unplanned urbanization leads increasing numbers of people to settle in neighborhoods with inadequate water, sanitation, and drainage infrastructure [9].

While diarrheal disease outbreaks in the wake of floods are regularly documented [7,16,17], and some context-specific studies have attributed these to individual pathogens [9,18,19], the precise causal pathways underlying these associations are not well understood. They are likely to be complex and time-dependent [6]. Different pathogen species and taxa possess their own distinct transmission dynamics and dominate or recede in importance as time elapses following the onset of a flood [7,20], highlighting the need to characterize impacts by etiological agent [6]. Furthermore, the relative roles of direct exposure to fecal contamination due to floodwaters compared to secondary effects of crowding and increased contact rates as a result of population displacement are not well delineated [21].

One approach to estimating the impact of flooding events on human health is to treat it as a ‘natural experiment’ by identifying disease surveillance data that spans the duration of the event as well as a sufficient pre- and post-flood comparison period providing a time series to which causal inference methods can be applied. Several studies using health information system data from China within case-crossover or interrupted time series (ITS) analyses have quantified significant increases in diarrheal disease and bacillary dysentery following floods in Anhui and Hunan provinces, respectively [22,23,24]. However, new diagnostic methods applied in multi-site, population-based studies now make it possible to characterize pathogen-specific trends in both diarrheal and asymptomatic enteric infections [25,26,27]. Using information from one such study, which happened to coincide with a La Niña-related flooding disaster, the objective of the analysis presented here was to use a comparative ITS analysis to derive causal inferences about the species-specific impacts of this event on enteric pathogen infections.

## 2. Materials and Methods

### 2.1. Study Setting

As part of the Etiology, Risk Factors and Interactions of Enteric Infections and Malnutrition and the Consequences for Child Health and Development (MAL-ED) project, data was collected from birth cohorts recruited from eight communities, each in a different low- or middle-income country [28]. Subjects were enrolled and monitored continuously over their first 2 years of life from November 2009 to March 2014. Written informed consent was obtained from the caregiver of all participating children.

**Figure 1:**
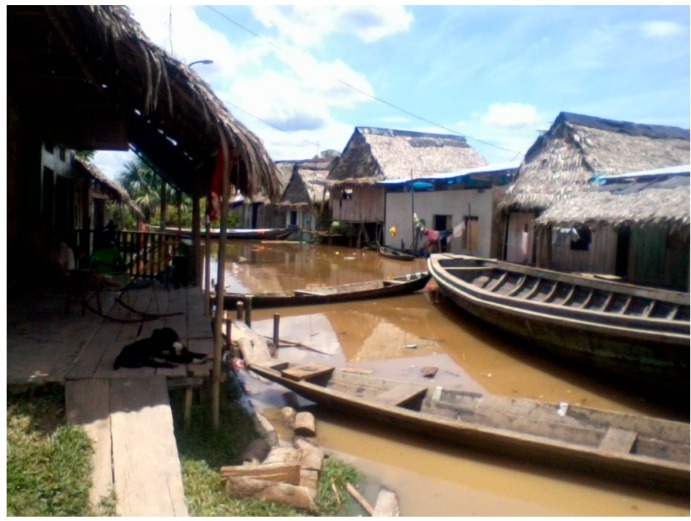
A flooded street in Santa Clara de Nanay, April 2, 2012 (courtesy of Asociación Benéfica Prisma).

**Figure 2:**
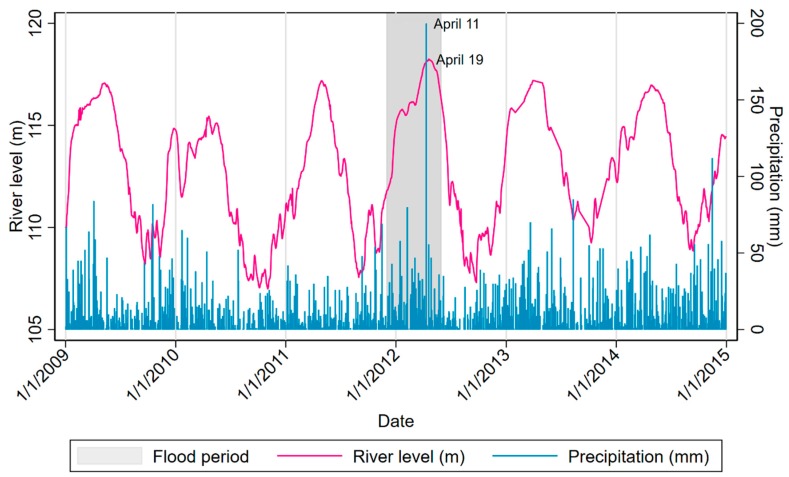
Daily precipitation volume measured at Coronel FAP Francisco Secada Vignetta International Airport 2009–2014 and levels of the Nanay River obtained from Sede Loreto at their intake station on the Nanay River [32,33].

One of the MAL-ED study sites is located in Santa Clara de Nanay, a peri-urban community located 15 km from the city of Iquitos in the Loreto province of Peru, in a low-lying equatorial rainforest area situated at the confluence of several Amazon tributaries [29]. Since waterways are the main transport infrastructure in this region, the large majority of the population is located on or in close proximity to the banks of rivers, making them particularly vulnerable to flooding. In late 2011 and early 2012, around midway through MAL-ED follow-up and under the strong influence of the 2010–12 La Niña event [10,11], the region was hit by widespread riverine floods after heavy rains over the preceding months caused the Ucayali, Marañón, and Nanay rivers to burst their banks [30,31]. In Santa Clara, torrential downpours began on November 15, 2011; and by December, around half of all households in the community had been displaced by flooding (Figure 1) and forced to evacuate their homes until March or April of 2012. The regional government of Loreto established temporary shelters for the displaced throughout the affected areas and, at the end of March of that year, declared the situation a state of emergency affecting over 191,000 inhabitants [31]. Heavy rains and the rise in river levels continued, on April 11 the weather station at the nearby airport recorded rainfall of 20 cm and by one week later, the Nanay River had risen to a level of 118.24 meters above sea level, its highest since 1986 (Figure 2) [32,33]. By the end of the disaster, an estimated 50,000 people had been made homeless [34].

### 2.2. Outcome Variables

Stool samples were collected from the subjects according to a predefined schedule at monthly intervals following enrollment in MAL-ED and upon reporting of a diarrheal episode by the child’s caregiver. For samples from subjects who completed follow-up, enteropathogen-specific infection status was ascertained using probe-based quantitative PCR (qPCR) assays on custom-developed TaqMan Array Cards (Thermo Fisher) [35]. For other samples, enzyme-linked immunosorbent assay (ELISA) was used to test for adenovirus, astrovirus, *Campylobacter*, *Cryptosporidum*, *Giardia*, and rotavirus, other bacteria species were assessed by culture with *E. coli* pathotypes confirmed by polymerase chain reaction (PCR), and norovirus was detected using reverse-transcription PCR [36]. The pathogen species included in this analysis were: adenovirus, astrovirus, norovirus, rotavirus, sapovirus, *Campylobacter*, enteroaggregative *E. coli* (EAEC), enteropathogenic *E. coli* (EPEC, typical and atypical), heat-labile enterotoxigenic *E. coli* (LT-ETEC), and heat-stable ETEC (ST-ETEC), *Salmonella*, *Shigella*/enteroinvasive *E. coli* (EIEC) (qPCR uses the same gene target for these two related pathogens), *Cryptosporidium*, and *Giardia*. To ensure that a single infection episode was not counted multiple times, *Campylobacter*- and norovirus-positive samples were excluded if they were collected within 30 days of a previous sample that was positive for the same pathogen strain without being separated by an intermediate negative sample. For all other pathogens, a 14-day period was used, except for the two protozoa, for which samples that were positive for the same species (*C. parvum* or *C. hominis*) or assemblage (*G. duodenalis* A or B) as a prior sample from the same subject were excluded unless separated by three negative samples.

### 2.3. Exposure Variables

The main exposure of interest—the ‘intervention’ in this ITS analysis—was whether the stool samples were collected during the approximate period of the flood. Two periods were considered and modeled separately using the same methods: the early flood period from December 1, 2011 to February 29, 2012, during which time the study community was flooded and many households were displaced; and the late flood period from March 1 to May 31, 2012, during which time evacuees returned to the community, but rains and flooding continued throughout the wider Loreto region. Sample-level binary dummy variables were therefore constructed that were coded as 1 if the sample was collected during the relevant flood period or 0 otherwise (the pre-/post-flood) [37]. The study therefore had a ‘BAB’ design (an inversion of the ‘ABA’ design as defined by Biglan and colleagues) since it includes the time before the intervention was introduced and the time after it was withdrawn [38]. Data from all eight sites were included in the analysis with the seven other sites used as a comparison group of concurrent reference populations to provide an external comparator to discount concurrent pandemic changes in incidence as alternative hypothesis to the observed differences during the risk period under evaluation in Loreto. A second binary dummy variable was therefore introduced corresponding to whether the subject was in the ‘treatment’ (Peru) or ‘control’ cohorts (the other seven sites) [37]. To adjust for background cyclical trends due to disease seasonality, terms for the interactions between annual and biannual Fourier-series sine and cosine functions terms and indicator variables for the eight sites were included in the model with the terms for the main effect omitted, thus allowing for up to two annual peaks and differences in their site-specific timing and magnitude [39,40]. In addition, the non-linear effect of age was modeled using linear, quadratic and cubic terms for the child’s age in continuous months. Study site, stool specimen type (diarrheal or surveillance) and diagnostic method (qPCR or conventional methods) were also adjusted for using indicator variables.

### 2.4. Statistical Methods

Modified Poisson regression models were fitted to each of the binary pathogen outcomes in turn using generalized linear models with cluster-robust variance estimation to calculate adjusted risk ratios (RRs) for infection [41]. The models assumed the following form (adapted from Linden and colleagues and Colston and colleagues [37,39]):

logit P(Yit=1)=β_0_+β_1_ T+β_2_ I_t_+β_3_ TI_t_+β_4_ Z+β_5_ ZT+β_6_ ZI_t_+β_7_ ZI_t_ T+⋯+β_n_ X_n_ (1)

where P(Yit = 1) is the probability of a stool from subject *i* being positive for pathogen *Y* on date *t*; *T* is the time in continuous months since the start of follow-up; *I_t_* is the dummy variable representing whether the intervention was in place at time *t* (i.e., whether date *t* took place between March and May of 2012); *Z* is the dummy variable denoting treatment or control cohort assignment; and *X_n_* are the covariates used for adjustment but not for estimating the main effect of the flood. In this equation, *β_6_* estimates the difference in the magnitude of the outcome variable immediately following the introduction of the intervention and can be interpreted as the difference in the RR of detection of pathogen *Y* at the start of the flood period compared with immediately before. *β_7_* represents the change in the outcome per unit of time while the intervention is in place—the trajectory of the relative risk during the flood. The estimates of *β_6_* and *β_7_* from each model were visualized in forest plots, and for pathogens for which these estimates were statistically significant at the *α* = 0.05 level, the trajectories in the probabilities of infection (calculated from the RRs estimated by the models) over the course of follow-up were plotted. As a secondary, exploratory analysis, detections of specific rotavirus genotypes were plotted in needle plots to visually assess differences in their timing relative to the flood periods. Analyses were carried out in Stata 15.1 (StataCorp, College Station, Texas, USA) [42].

## 3. Results

Table 1 shows the distribution of positive detections of the different species of enteropathogens in stool samples from the MAL-ED Peru site during early and late flood periods and the pre-/post-flood period, as well as their overall distribution in samples from the other seven sites. In all periods and in both treatment and control group, the most prevalent pathogen was EAEC followed by *Campylobacter*. Norovirus, sapovirus, and *Giardia* were also highly prevalent. The needle plots in Figure S1 in the supporting information show the daily distribution of pathogen-positive stool samples recorded at the Peru site by species.

**Figure 3:**
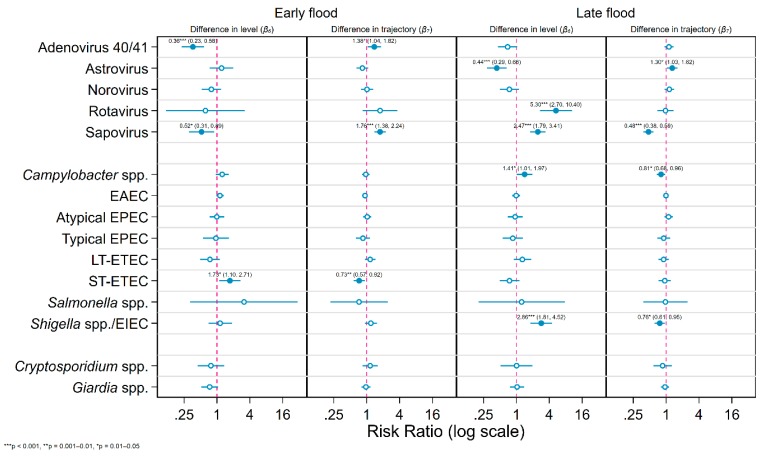
Risk ratios for detection of specific enteric pathogen species in stool samples collected from infants aged 0–2 years during each of the two flood periods relative to the pre-/post-flood period and to the control sites estimated for generalized linear models that adjusted for seasonality, site, age, sample type, and diagnostic method.

The forest plots in Figure 3 visualize the risk ratios and their confidence intervals and significance levels estimated by the two models for the detection of each enteric pathogen species in the two flood periods relative to the pre-/post-flood period and to the control sites adjusted for confounders. During the early period of the flood, ST-ETEC was the only pathogen for which an increased risk was observed—a slightly statistically significant 73% increase (RR = 1.73 [1.10, 2.71]) relative to the non-flood period and control group, which decreased by 27% per month following the start of that period (RR = 0.73 [0.57, 0.92]). Adenovirus exhibited a highly and sapovirus a moderately statistically significant decrease in risk during the early flood period (RRs respectively 0.36 [0.23, 0.58] and 0.52 [0.31, 0.89]). During the later period of the flood, the largest RR of any of the pathogens was observed for rotavirus, an increase in risk of over 500% relative to the pre-/post-flood period and control group (RR = 5.30 [2.70, 10.40]). Risk of sapovirus also increased substantially and highly statistically significantly by almost 250% (RR = 2.47 [1.79, 3.41]), while a highly statistically significant decrease in astrovirus risk was observed (RR = 0.44 [0.29, 0.66]). Among the enteric bacteria, the largest effect size was seen for *Shigella* spp., risk of which almost tripled in the late flood period compared to the non-flooded period and control group (RR = 2.86 [1.81, 4.52]), while a slightly statistically significant increase in the risk of *Campylobacter* spp. was also observed (RR = 1.41 [1.01, 1.97]). No associations with either of the two protozoa were significant at the α=0.05 level, although a decrease in risk of *Giardia* spp. during the early flood period was borderline significant. Pathogens with statistically significant differences in levels in the intervention periods tended to have differences in trajectory of corresponding significance and magnitude in the other direction, indicating that changes in transmission reverted to background levels relatively promptly (the notable exception being rotavirus).

**Figure 4:**
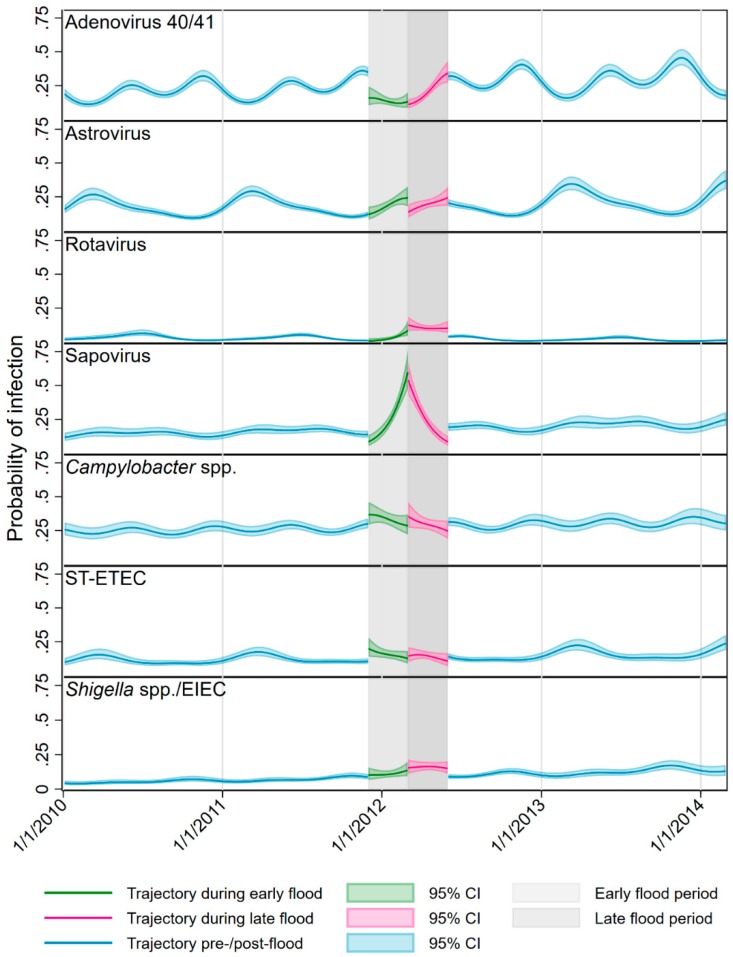
Transmission trajectories predicted by the models with (confidence intervals—CIs) for the seven pathogens that exhibited statistically significant effects (probabilities calculated from relative risk estimates).

Figure 4 shows the trajectories predicted by the models over the course of follow-up after the RRs were converted to probabilities for the seven pathogens for which statistically significant effects were identified. For adenovirus the models predicted biannual seasonality in transmission at the Peru site with a primary peak in November and a secondary, mid-year peak. The flood started during the primary peak and an immediate decrease in the probability of adenovirus was detected, which persisted into the late flood period, but had returned to pre-flood levels by the end of May 2012. Astrovirus exhibited a single annual peak occurring early in the year. This pattern did not appear to be disrupted in the early flood period, but in the later period, the probability of infection decreased substantially, before returning to pre-flood levels. Compared with other viruses, rotavirus transmission at this site was low (Peru introduced the Rotarix vaccine in 2008 [29]), with a single midyear peak. During the year of the flood, probability of infection rose early and was sustained through the late flood period at a level higher than the normal seasonal peak. Sapovirus did not demonstrate marked seasonality, but probability of infection rose to several times the normal level over the course of the early flood period and decreased to a lower level than normal in the late period. The trend for *Campylobacter* showed low-amplitude, biannual seasonality with upticks at the very start of both the early and late flood periods. ST-ETEC had a single annual peak in the first quarter of the year and also showed evidence of an off-peak uptick at the start of the early flood period, with trends during the late flood returning to approximately the pre-/post-flood pattern. Transmission of *Shigella* spp./EIEC showed little seasonal variation, rose over the early flood period and had not returned to normal levels by the end of the late flood period.

**Figure 5:**
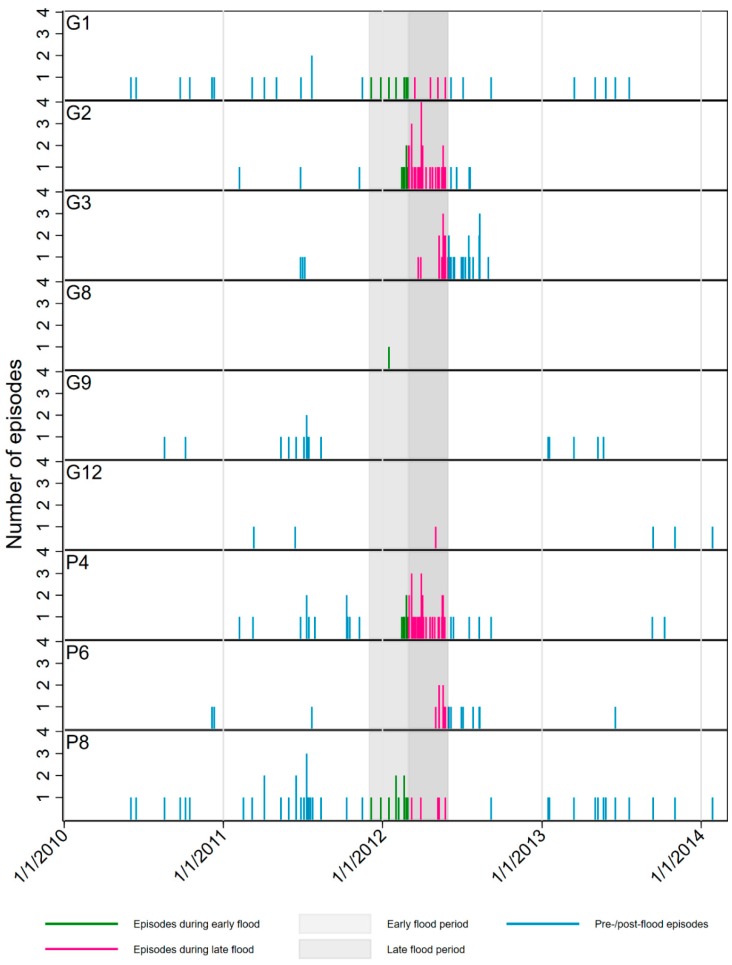
Needle plots of the daily distribution of rotavirus-positive stool samples recorded at the MAL-ED Peru site by genotype.

The needle plots in Figure 5 show the daily distribution of rotavirus-positive stool samples recorded at the Peru site by genotype. While G1 and P[8] types occurred evenly and G8, G9, and G12 sporadically throughout the follow-up period, the majority of G2 and P4 rotavirus episodes were recorded in a cluster starting at the end of the early flood period and continuing beyond the end of the late flood period. A similar, but less pronounced cluster of G3 and P6 rotavirus episodes began towards the end of the late flood period.

## 4. Discussion

In order for health systems to adapt to mitigate the impacts of climate change, evidence is needed of the impacts of extreme weather events on specific outcomes of public health importance. The El Niño–Southern Oscillation (ENSO) phenomenon is a cause of both flooding and droughts across multiple continents and has been shown to be associated with increases in childhood diarrhea in Peru, the United States and elsewhere [43,44,45]. Climate change may increase the frequency of ENSO events as well as the global land area that is subject to its precipitation impacts [46,47]. Evidence regarding the effects of flooding on individual diarrhea-causing pathogens is limited, but is urgently needed in order to plan and implement interventions and prioritize resources before climate-related disasters strike. This study applied a causal inference approach to data collected from infants enrolled in a multisite study that deployed broadly inclusive diagnostics for common enteropathogens that are among those most closely linked to cases of moderate and severe diarrhea [26,48]. In doing so, statistically significant increases in the prevalence of several important pathogens during the course of the flood were identified—including rotavirus, sapovirus, ST-ETEC, and *Campylobacter* spp. and *Shigella* spp.—strongly suggesting that the transmission of these pathogens are most sensitive to flooding in this context. Numerous other pathogens did not show statistically significant effects, indicating that they may not be responsive to floods, findings which, if replicated elsewhere, may mean that they can be deprioritized in disaster preparedness policies.

Risk of ST-ETEC infection in the cohort was elevated during the first three months of the flood period, but not during the later phase. ETEC is easily transmitted in contaminated water and frequently detected in surface water in riverine settings, environments where it is adapted to survive by upregulating certain genes involved in membrane stability and by forming biofilms [49,50]. Evidence from successive floods in Bangladesh strongly implicate these events as major drivers of ETEC diarrhea, though the dominance of the ST-relative to the LT-producing form appeared to vary [17,21,49]. Elevated risk of infection with *Campylobacter* spp. and *Shigella* spp./EIEC—both bacterial enteropathogens with low infectious doses—were identified in the late flood period. *Campylobacter* is a zoonotic bacterium for which poultry is a primary reservoir and can survive in surface waters and aquatic environments [51,52]. Observed increases in the incidence of campylobacteriosis following extreme precipitation events are thought to be due, at least in part, to the contamination of water sources by runoff from concentrated animal feeding operations (CAFO) [53]. It is possible that the results from Santa Clara represent a smaller-scale version of this process, occurring in a community with high rates of household chicken ownership.

While no significant difference in rotavirus prevalence was observed during the first three months of the flood, the late flood period saw a five-fold increase in rotavirus infection risk, the largest relative effect identified by this analysis. While the primary routes of rotavirus transmission involve direct person-to-person transmission or contact with contaminated surfaces and fomites, the importance of waterborne transmission is gaining salience [54]. Flooding in the Solomon Islands in 2014 caused a nationwide outbreak of rotavirus diarrhea [55], while recent evidence from Bangladesh have linked both particular precipitation events—notably the 2007 flood [17]—and rainy days in general to upticks in rotavirus [56]. Previous analyses of data from the South Asian MAL-ED sites have identified secondary seasonal peaks in rotavirus coinciding with the annual monsoon season [39,57] and recent findings of a protective effect of drinking water from tube wells are consistent with these results, since these water supply systems draw upon deeper groundwater reserves that are better protected from flood-related contamination than sources nearer to the surface [58]. Mechanistic simulation modeling has demonstrated that dissemination of rotavirus between communities connected by waterways can be an important indirect route of transmission in tropical environments that is modified by flow velocity [54]. It is possible that differences in the flow rate of the floodwaters may explain why an effect was observed in the late but not the early flood period.

The exploratory analysis of the epidemiology of the specific rotavirus genotypes at the Peru MAL-ED site suggest that a small outbreak of G2P[4] type virus occurred starting in late February 2012, with a smaller outbreak of G3P[6] type starting towards the end of the late flood period. Although sporadic identifications did occur outside of the flood period, these are generally atypical genotypes in this setting, where G1 and P[8]—the combination that are the target of the Rotarix vaccine used at this site—are by far the dominant circulating genotypes. Indeed, a later cohort study recruited from the same community in 2018 (a year in which no substantial flooding occurred), carried out during the local annual rotavirus peak found only G1 and P[8] genotypes in circulation (unpublished results). A possible explanation for these findings is that as a result of the flood, locally atypical, non-vaccine rotavirus strains were introduced to the community—either carried along the waterways by floodwater from upstream communities, or else brought by returning evacuees acting as hosts—which then briefly took hold. While estimates of Rotarix efficacy against G2P[4] vary, they are generally lower than for the target genotype (41% compared to 92% in one study [59] 85% compared to 95% in another [60]) suggesting that there would have been higher susceptibility to an allochthonous introduction of that strain. Although secular changes and reassortments unrelated to flood are known to occur following vaccine introduction [61,62] and cannot be ruled out in this case, the fact that the emergence of G2P[4] rotavirus at this location occurred outside of the normal peak season and in conjunction with a similar appearance of the more unusual G3P[6] reassortant suggest that influences outside of the normal dynamics were at play. The appearance of genotypes with a recognized lower, but still significant vaccine efficacy suggests that rotavirus disease would likely have been significantly greater in the absence of immunization. This outbreak may also explain why overall rates of rotavirus transmission were unusually high at the MAL-ED Peru site in spite of high levels of vaccine uptake and compliance [63]. Modeling the risk ratios for specific genotypes in the same way as the main pathogen species in most cases yielded effect estimates that were so large as to be ungeneralizable outside of this very specific context.

Waterborne transmission occurs as a secondary route of transmission for all the other enteric viruses and both adenovirus and norovirus have been implicated in the scientific literature in outbreaks following extreme water-related weather events [64]. The most striking effect in absolute terms identified in this analysis was the sharp, off-season spike in sapovirus transmission that occurred around midway through the overall flood period. Historically, sapovirus has been underexplored relative to other enteric pathogens, so there is correspondingly little precedent for these findings in the scientific literature [65], and this study contributes to a growing body of evidence of the need for more population-based research into the epidemiology of this virus. The decreased risk of adenovirus in the early and astrovirus in the late flood periods are notable insofar as both occurred during the primary local annual peak in their transmission, suggesting that floodwaters may wash them from the environment, disrupting their seasonal trends [6].

This study was subject to several limitations. Given the restricted age range of subjects enrolled in MAL-ED, the study only identified infections in infants, while those occurring in later childhood, adolescence, or adulthood went undetected. Transmission patterns in response to flooding events may differ in older age groups. Furthermore, it was not possible to test specific hypotheses about the particular transmission pathways, since these may be pathogen-specific and require more resource intensive methods. Future research in this area may employ microbial source tracking to validate hypotheses about the relative contribution of humans compared to animals as pathogen reservoirs and clarify transmission routes [66].

These findings have several implications for policy-makers wishing to undertake preemptive strategies to reduce the risk of enteric disease outbreaks due to flooding. Firstly, while high vaccine coverage is necessary to sustain decreases in background rotavirus transmission levels, it may not prevent the local introduction of non-vaccine virus strains due to exogenous events such as flooding. More sustainable protection may be afforded by providing water sources that rely on groundwater reserves that are more resilient against viral contamination than surface sources [58]. Communities where household ownership of livestock is common may be at particular risk, suggesting that, community-level sustainable animal manure management interventions may prevent environmental contamination from livestock waste [67]. Lastly, in the context of three recent trials of water, sanitation, and hygiene (WASH) interventions that found at best only qualified impacts on diarrheal disease outcomes, this study adds further evidence to calls for a more radical, transformative WASH agenda [68]. Traditional low-cost, household-level improvements to water sources and sanitation facilities of the kind provided in such trials and by which progress towards WASH targets are measured, may simply be inadequate in the face of climate events that may suddenly and unexpectedly expose entire communities to large amounts of untreated sewage. Investments in more ambitious, municipal-level water, wastewater, and drainage infrastructure of the kind that have historically engendered society-wide child health improvements when implemented in high income countries may be the only sure route to climate resilience, if properly adapted to local contexts [68].

## 5. Conclusions

Causal inference approaches, such as interrupted time series, can be applied to population health surveillance data to shed light on the mechanisms behind disease transmission and quantify the effects of natural disasters. Using these methods, this analysis found that floods related to the La Niña phenomenon were associated with statistically and clinically significant increases in the risk of infection of two enteric viruses (rotavirus and sapovirus) and three enteric bacteria ( *Campylobacter* spp., ST-ETEC, and *Shigella* spp.) after controlling for potential sources of bias and confounding. Policy-makers should target interventions towards these pathogens—including vaccines as they become available—in settings where vulnerability to flooding is high as part of disaster preparedness strategies. More generally, investments in radical, transformative, community-wide, and locally-tailored water and sanitation interventions are needed to ensure the resilience of vulnerable populations against the health impacts of extreme rainfall events.

## Figures and Tables

**Table 1:** Number and percentages (%) of stool samples that were positive for different species of enteropathogens in the MAL-ED Peru site during three periods relative to the flood and in the other seven study sites (control group) overall.

Pathogen	Early Flood	Late Flood	Pre-/Post-Flood	Control Group
Adenovirus 40/41	77 (9.0)	129 (15.4)	1171 (18.3)	4545 (10.9)
Astrovirus	114 (13.5)	121 (14.5)	859 (13.4)	3800 (9.1)
Norovirus	96 (13.6)	135 (18.5)	1189 (21.6)	6002 (15.8)
Rotavirus	25 (2.9)	85 (10.2)	173 (2.6)	2027 (4.8)
Sapovirus	119 (20.5)	122 (20.3)	689 (15.4)	4693 (13.3)
*Campylobacter* spp.	202 (24.4)	194 (23.7)	1,386 (22.3)	10,248 (25.6)
EAEC	359 (47.1)	379 (48.8)	2,585 (43.3)	17,414 (42.6)
Atypical EPEC	176 (21.3)	162 (20.0)	1,212 (19.2)	8,593 (20.5)
Typical EPEC	70 (8.4)	97 (11.9)	622 (9.7)	4344 (10.4)
LT-ETEC	113 (13.5)	132 (16.2)	918 (14.4)	4766 (11.4)
ST-ETEC	88 (10.6)	80 (9.7)	584 (9.1)	5,397 (12.9)
*Salmonella* spp.	7 (0.8)	10 (1.1)	50 (0.7)	264 (0.6)
*Shigella* spp./EIEC	86 (9.5)	125 (14.4)	606 (9.0)	4126 (9.8)
*Cryptosporidium* spp.	78 (9.4)	44 (5.3)	507 (8.1)	2183 (5.3)
*Giardia* spp.	125 (17.4)	156 (22.4)	1117 (20.8)	5998 (16.7)

## Supplementary Materials

The following are available online at https://www.mdpi.com/1660-4601/17/2/487/s1, Figure S1: Needle plots of the daily distribution of pathogen-positive stool samples recorded at the MAL-ED Peru site by species.
