# Supplementary material for: Pathogen-Specific Impacts of the 2011–2012 La Niña-Associated Floods on Enteric Infections in the MAL-ED Peru Cohort: A Comparative Interrupted Time Series Analysis
Source: Int J Environ Res Public Health. 2020 Jan 12;17(2):487. doi: 10.3390/ijerph17020487 (PMC7013961; doi:10.3390/ijerph17020487)
Supplement: Supplementary file 1 [file ijerph-17-00487-s001.pdf]

International Journal of *Environmental Research and Public Health*

MDPI

Article

Pathogen-Specific Impacts of the 2011-12 La Niña-Associated Floods on Enteric Infections in the MAL-ED Peru Cohort: A Comparative Interrupted Time Series Analysis

Josh Colston [1], Maribel Paredes Olortegui [2], Benjamin Zaitchik [3], Pablo Peñataro Yori [4], Gagandeep Kang [5], Tahmeed Ahmed [6], Pascal Bessong [7], Esto Mduma [8], Zulfiqar Bhutta [9], Prakash Sunder Shrestha [10], Aldo Lima [11] and Margaret Kosek *

[1] Division of Infectious Diseases and International Health, University of Virginia School of Medicine, city, post code, VA, USA josh.colston@virginia.edu
[2] Asociación Benéfica Prisma, Iquitos, post code, Peru, mparedeso@prisma.org.pe
[3] Department of Earth and Planetary Sciences, Johns Hopkins Krieger School of Arts and Sciences, city, post code, MD, USA, zaitchik@jhu.edu
[4] Division of Infectious Diseases and International Health, University of Virginia, Charlottesville, post code, VA, USA, pyori@virginia.edu
[5] Christian Medical College, Vellore, post code, India, gkang@cmcvellore.ac.in
[6] Nutrition & Clinical Services Division, International Centre for Diarrhoeal Disease Research, Bangladesh (icddr,b), Dhaka, post code, Bangladesh, tahmeed@icddrb.org
[7] University of Venda, Thohoyandou, post code, South Africa, Pascal.Bessong@univen.ac.za
[8] Haydom Global Health Institute, Haydom, post code, Tanzania, estomduma@gmail.com
[9] Department of Pediatrics and Child Health, Aga Khan University, Karachi, post code, Pakistan, zulfiqar.bhutta@aku.edu
[10] Department of Child Health, Institute of Medicine of Tribhuvan University, city, post code, Nepal, prakashsunder@hotmail.com
[11] Federal University of Ceará, Fortaleza, post code, Brazil, alima@ufc.br
* Correspondence: mkosek@virginia.edu;

Commented [m1]: Please carefully check the accuracy of names and affiliations.

Commented [m2]: Please confirm the Affiliation of Prof. Margaret **Kosek.**

Commented [m3]: Please add city and post code

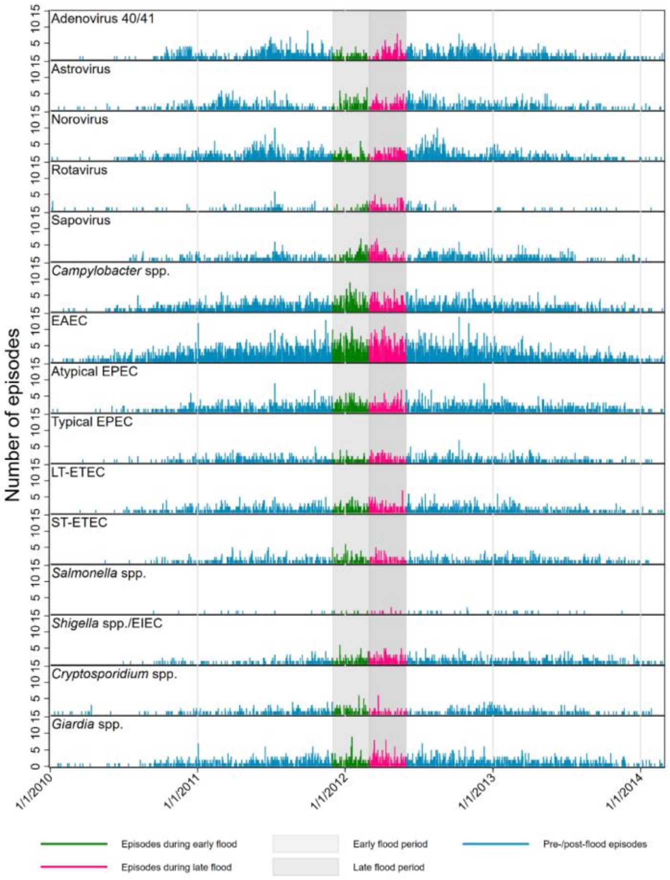

Figure S1. Needle plots of the daily distribution of pathogen-positive stool samples recorded at the MAL-ED Peru site by species.

© 2020 by the authors. Submitted for possible open access publication under the terms and conditions of the Creative Commons Attribution (CC BY) license (http://creativecommons.org/licenses/by/4.0/).
